# Abnormal spontaneous activity and rest–task shift in schizophrenia

**DOI:** 10.1111/pcn.13880

**Published:** 2025-08-12

**Authors:** Ryo Mitoma, Shunsuke Tamura, Shogo Hirano, Yubin Sung, Yoshifumi Takai, Takako Mitsudo, Tomohiro Nakao, Toshiaki Onitsuka, Yoji Hirano

**Affiliations:** ^1^ Department of Neuropsychiatry, Graduate School of Medical Sciences Kyushu University Fukuoka Japan; ^2^ Department of Psychiatry, Division of Clinical Neuroscience, Faculty of Medicine University of Miyazaki Miyazaki Japan; ^3^ Division of Clinical Research National Hospital Organization, Hizen Psychiatric Center Saga Japan; ^4^ NHO Sakakibara National Hospital Tsu Japan; ^5^ Institute of Industrial Science The University of Tokyo Tokyo Japan

**Keywords:** abnormal rest–task shift, alpha and gamma oscillation, auditory steady‐state response (ASSR), schizophrenia, spontaneous activity

## Abstract

**Aims:**

Schizophrenia (SZ) is associated with abnormalities in both spontaneous and task‐evoked neural oscillations, and growing evidence shows that shift patterns of oscillatory activity between resting and task states are also disturbed. However, no study has simultaneously examined the frequency‐ and state‐specific characteristics of oscillatory deficits in SZ. Using an auditory steady‐state response (ASSR) paradigm, we aimed to examine the differential sensitivity of oscillatory measures to SZ and to assess rest–task shifts across multiple frequency bands.

**Methods:**

We recorded resting‐state activity and 40 Hz ASSR of 66 neurotypical controls (NC) and 68 SZ patients using electroencephalography (EEG). 40 Hz stimulus‐evoked activity was measured using evoked power, phase‐locking factor (PLF), and phase‐locking angle, whereas multi‐frequency (4–100 Hz) spontaneous activity during ASSR and resting states was assessed using induced and resting power. The state‐dependent shifts in spontaneous activity between the resting and ASSR states were evaluated over a broad frequency range.

**Results:**

Both induced and resting power in the low‐frequency range (4–10 Hz) were elevated over widespread regions in SZ patients relative to NC. Gamma‐band (39–100 Hz) induced power then demonstrated excellent ability to discriminate between SZ and NC. In addition, SZ patients showed a reduced rest–task shift in the theta‐beta band (5–23 Hz) spontaneous power, most pronounced in the alpha‐band (8–13 Hz).

**Conclusion:**

The present study confirmed the utility of gamma‐band induced power during ASSR stimulation for differentiating SZ patients from NC. Importantly, our results also highlight the pathophysiological significance of the reduced rest–task shift pattern of spontaneous activity mainly in the alpha‐band in SZ patients.

Accurate temporal coordination of brain activity through neural oscillations is essential for efficient information processing.^1^, ^2^ Among the multiple frequency bands of neural oscillations (delta‐band: 1–4 Hz; theta‐band: 4–8 Hz; alpha‐band: 8–13 Hz; beta‐band: 13–30 Hz; gamma‐band: 30–100 Hz), gamma‐band activity is responsible for the temporal coordination of localized neural circuits.^3^, ^4^, ^5^ The generation of gamma‐band activity requires an appropriate balance between excitatory and inhibitory neurons (E/I balance), where the excitatory output from pyramidal cells is precisely inhibited by fast‐spiking inhibitory interneurons.^5^, ^6^, ^7^ Therefore, gamma‐band oscillatory activity is hypothesized to reflect the altered E/I balance in various neuropsychiatric disorders.^8^, ^9^, ^10^

Schizophrenia (SZ) is a debilitating psychotic disorder with symptoms such as hallucinations, delusions, and cognitive and affective deficits. Accumulating evidence indicates that neural oscillatory abnormalities in SZ have been observed in a broad frequency range during both resting and task states.^11^ Reflecting different cognitive roles across multi‐frequency bands,^1^, ^12^ these abnormalities have been linked to various psychopathological and cognitive symptoms of SZ.^11^, ^13^ Moreover, there has been growing recognition that a disruption in the state‐dependent modulation of oscillatory activity may represent another pathophysiological feature of SZ. Specifically, a recent comprehensive review proposed that aberrant control of spontaneous activity could cause reduced differentiation of neural activity between resting and task states (i.e. reduced ‘rest–task shift’), and this abnormal shift may also relate to the clinical manifestations of SZ.^14^

Disturbances in gamma‐band oscillations have attracted attention as a potential pathophysiological mechanism of SZ because their relationship with clinical symptoms (e.g. hallucinations) in SZ patients has been repeatedly reported.^15^, ^16^, ^17^ To assess gamma‐band deficits in SZ, previous studies have evaluated gamma‐band stimulus‐evoked or spontaneous activities using electroencephalography (EEG)^18^, ^19^ or magnetoencephalography (MEG).^20^, ^21^, ^22^ The most commonly evaluated parameter for gamma‐band stimulus‐evoked deficits in SZ was the auditory steady‐state response (ASSR), a sustained response elicited by click trains or amplitude‐modulated sounds.^23^, ^24^ Numerous ASSR studies have reported a decrease in the gamma‐band phase‐locking factor (PLF) (the degree of stimulus phase locking) and evoked power, which measure stimulus‐evoked activity, in patients with chronic^25^, ^26^, ^27^ and first‐episode SZ.^28^, ^29^ Meta‐analyses^30^, ^31^ and a scoping review^32^ suggest that these gamma‐band ASSR measures are robust findings in SZ. In addition, a novel ASSR measure for detecting altered gamma‐band stimulus‐evoked activity in SZ has been introduced to potentially complement PLF and evoked power. Roach *et al*.^33^, ^34^ reported that gamma‐band phase delay in 40 Hz ASSRs, quantified by phase‐locking angle (PLA), had greater differential sensitivity to SZ and better test–retest reliability than did PLF and evoked power. It has been suggested that this phase delay may be more sensitive than amplitude‐ or synchrony‐based measures to timing‐specific pathophysiological mechanisms of gamma‐band oscillations (e.g. altered E/I balance).^33^

In addition to gamma‐band ASSR activity, resting‐state activity and non‐phase‐locked activity during ASSR stimulation have been evaluated to identify gamma‐band spontaneous deficits in SZ.^35^, ^36^, ^37^, ^38^ As highlighted by a recent meta‐analysis,^39^ findings on gamma‐band resting power in SZ remain inconclusive: while several studies have reported increased power,^36^, ^40^ others have indicated decreased power^41^, ^42^ or no significant differences from neurotypical controls (NC).^23^, ^43^ In contrast, the non‐phase‐locked power during ASSR stimulation (referred to as ‘induced power’) has consistently been reported as greater in SZ patients than in NC.^23^, ^35^, ^44^

Despite substantial evidence of gamma‐band deficits in SZ patients, the optimal measure for detecting altered gamma‐band activity during resting‐state or ASSR stimulation in SZ patients has yet to be established. It should also be noted that an exclusive focus on gamma‐band activity may obscure other pathology‐relevant abnormalities in spontaneous oscillatory dynamics. Accumulating evidence from resting‐state EEG studies indicates elevated delta‐ and theta‐band activity in SZ, accompanied by a robust reduction in alpha‐band activity.^39^, ^45^ In contrast, findings regarding beta‐band in the resting‐state activity remain inconsistent.^45^ With respect to induced power, to the best of our knowledge, no studies have investigated frequency bands beyond the gamma range in SZ. Considering that induced power reflects spontaneous activity during the task state, evaluating induced power across multiplefrequency bands may offer a more comprehensive understanding of neural oscillatory alterations in SZ. Moreover, comparing the resting power and induced power is suitable for evaluation of the rest–task shift because both of them capture spontaneous activity through the same measure. Nonetheless, to date, no ASSR studies have specifically investigated rest–task shift dysfunction in spontaneous activity among SZ patients.

Building on this background, our study had two principal objectives. The first was to explore which oscillatory measures of stimulus‐evoked and spontaneous activity show the greatest differential sensitivity to SZ pathology. The second was to investigate which frequency bands exhibit reduced rest–task shifts of spontaneous activity between the ASSR and resting states in SZ patients. Specifically, we compared the amount of change in spontaneous activity between the two states across multiple frequency bands. To achieve these objectives, we assessed stimulus‐evoked and spontaneous activities during the resting and ASSR states in a large sample of SZ patients. This research was conducted as part of the Asian Consortium on EEG Studies in Psychosis (ACEP, http://acep.saturn.bindcloud.jp), and it marks the first step toward enabling high‐quality, multifacility collaborative studies using the same clinical EEG systems.

## Methods

### Subjects

Sixty‐six neurotypical controls (NC) (mean age: 37.0 ± 10.3 years, 30 males) and 68 SZ patients (mean age: 34.9 ± 12.3 years, 35 males) participated in the experiment. SZ patients were recruited from Kyushu University Hospital, and NC were recruited from the local community in the Fukuoka metropolitan area. The exclusion criteria included age >60 years, neurological illness, major head trauma, history of electroconvulsive therapy, substance abuse and verbal IQ <75. All the NC were screened using the Structured Clinical Interview (non‐patient edition), and no controls or their first‐degree relatives were found to have an Axis I psychiatric disorder. Two experienced psychiatrists (YH and TO) diagnosed SZ patients in accordance with the Diagnostic and Statistical Manual of Mental Disorders, 5th Edition (DSM‐5). Symptom severity was assessed using the Positive and Negative Syndrome Scale (PANSS),^46^ and its five‐factor scores (positive, negative, disorganized/concrete, excited, and depression) were obtained by summing the PANSS items assigned to each factor.^47^ The demographic and clinical characteristics of these subjects are summarized in Table 1. The study was approved by the Kyushu University Institutional Review Board for Clinical Trials (approval number 20192034) and conformed to the latest version of Declaration of Helsinki. Written informed consent was acquired from each subject after providing a detailed explanation of the study.

**Table 1:** Demographic and Clinical Characteristics of Subjects

Variable^†^	NC (*n* = 66)	SZ (*n* = 68)	Statistic	*P*
Age, years^‡^	36.95 (10.26)	34.94 (12.26)	*t*(129.22) = 1.03	0.30
Sex: Male/Female, *n* ^§^	30/36	35/33	χ^2^(1) = 0.49	0.49
Handedness: Left/Right/Both, *n* ^§^ ^,^ ^¶^	2/59/0	3/57/1	χ^2^(2) = 1.23	0.54
Education, years^‡^ ^,^ ^††^	15.40 (2.78)	13.54 (2.61)	*t*(121.66) = 3.88	<0.01
Duration of illness, years		8.32 (9.76)		
Medication dosage (CPZ equiv., mg)^‡‡^		514.57 (460.69)		
PANSS five‐factor scales				
Positive		12.22 (5.71)		
Negative		21.07 (8.86)		
Disorganized/Concrete		14.46 (7.22)		
Excited		10.01 (5.23)		
Depression		14.26 (6.53)		

^†^ Mean (SD) values are given for each variable.

^‡^ Statistical analysis was performed by Welch's *t* test.

^§^ Statistical analysis was performed by chi‐square test.

^¶^ There were missing data for five neurotypical controls and seven schizophrenia patients.

^††^ There were missing data for six neurotypical controls.

^‡‡^ There were missing data for four patients.

CPZ, chlorpromazine; NC, neurotypical control; PANSS, positive and negative syndrome scale; SZ, schizophrenia.

### EEG recordings and preprocessing

The Neurofax EEG system (EEG‐1200; Nihon‐Kohden Co.) was used to record EEG signals at a sampling rate of 500 Hz using a 19‐electrode silver/silver chloride (Ag‐AgCl) passive electrode based on the standard 10–20 system.^48^ Each electrode was referenced to the ipsilateral earlobe (A1 left, A2 right). For the assessment of eye movements, additional electrodes were attached to the outer canthi and the supraorbital foramen of the right eye. The impedance of all electrodes was less than 20 kΩ.

**Fig. 1:**
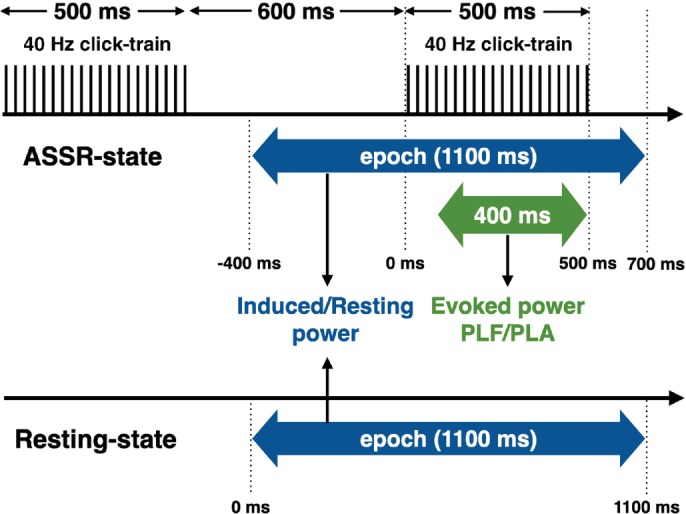
Schematic illustration of the electroencephalography (EEG) recording procedure. Resting‐state EEG recording of more than 3 min was followed by an auditory steady‐state response (ASSR) test, consisting of 150 stimulations of 40 Hz click trains with a 500 ms duration and a 600 ms interstimulus interval. The ASSR and resting states EEG data were subdivided into epochs with a duration of 1100 ms. For the ASSR‐state epochs, the period of 100–500 ms after stimulus onset was used for analysis of evoked power, phase‐locking factor (PLF), and phase‐locking angle (PLA).

To evaluate stimulus‐evoked and spontaneous activities, we recorded the resting‐state EEG signals (mean: 256 ± 76 s) followed by the ASSR‐state EEG signals (Fig. 1). For the ASSR stimulation, 40 Hz trains of 1 ms white noise clicks with a 500 ms duration were presented 150 times, each with a 600 ms interstimulus interval, through inserted earphones (ER‐3, Etymotic Research, Inc.). The sound pressure level was set to 80 dB using a sound level meter (type 2260; Brüel and Kjær), a 1/2‐in. condenser microphone (type 4192; Brüel and Kjær), and a custom‐made coupler (AD‐0213; Kyushu InterTech). During both the ASSR and resting states recordings, participants were instructed to rest in a supine position with their eyes closed in a sound‐attenuated and magnetically shielded room. EEG data were analyzed with MNE Python (https://mne.tools/dev/index.html). An offline 1–100 Hz bandpass filter and notch filters of 60 Hz were applied to the raw EEG data. Before the epochs were created, eye blinks, vertical and horizontal eye motions, and cardiac and muscle artifacts were removed *via* independent component analysis from raw data. Artifact‐free raw data were average referenced, and epochs of 1100 ms were created for each state separately. Epochs in the ASSR‐state started at 400 ms prior to stimulus onset (referred to as ‘−400 ms’) and lasted until 700 ms after stimulus onset. The resting‐state epochs were created by segmenting the resting‐state EEG data every 1100 ms with no overlap. After rejecting epochs with peak‐to‐peak amplitudes >200 μV, more than 120 epochs were accepted in both the ASSR and resting states for all participants. To avoid the adverse effects of differences in the number of accepted epochs on the subsequent analyses, 120 epochs were randomly selected without replacement from the accepted epochs in each state. For the ASSR‐state, we obtained the stimulus‐evoked data by averaging selected epochs across trials.

### EEG analysis

The measures of stimulus‐evoked activity (i.e. PLF, evoked power, and PLA) were obtained at all electrodes in the time‐frequency domain (time range, −400 to 700 ms; frequency range, 4–100 Hz with 1 Hz bins) by applying Morlet wavelet transforms to each epoch and stimulus‐evoked data in the ASSR‐state. The procedures for obtaining each measure at each electrode are described below. Evoked power is the spectro‐temporal power of stimulus phase‐locked activity and was calculated by applying time–frequency analysis to the evoked response. Baseline correction was performed at each frequency by subtracting the time‐averaged evoked power during the prestimulus period (−100 to 0 ms) from evoked power at each time point. PLF measures the degree of phase synchronization across trials and ranges from 0 (random distribution) to one (perfectly phase locked). The detailed procedure for obtaining PLF at each time–frequency point was as follows: the norm of the complex vector obtained by applying time–frequency analysis to each epoch was standardized to one, and PLF was defined as the norm of the averaged complex vector across trials. The PLA was defined as the degree to which a participant's oscillatory phase leads or lags relative to the expected angles (averaged angles of the NC participants).^33^ The individual angle at each time–frequency point was determined from the trial‐averaged complex vector obtained through PLF calculations.

Induced and resting power were computed as mean power spectral density (PSD) across epochs during ASSR or resting states. PSDs were calculated separately for each electrode in the 4–100 Hz frequency range using a multi‐taper method,^49^ with a half‐bandwidth of four for the multi‐taper window. To calculate induced power, we first obtained non‐phase‐locked responses during ASSR stimulation by subtracting the stimulus‐evoked data from each epoch to isolate the induced activity.^50^ PSDs were then computed for each epoch of the non‐phase‐locked response and subsequently averaged across trials. In contrast, resting power was obtained by computing PSDs for each resting‐state epoch and averaging the results across epochs.

### Statistical analysis

Before subsequent analyses, we calculated the mean PLF and evoked power within the time window (100–500 ms) and frequency range (40 Hz: 38–42 Hz) at all electrodes. The mean PLA at 40 Hz over the same time window (100–500 ms) was also calculated. For both induced and resting power, we used the PSD values over all frequencies (4–100 Hz) for statistical analysis. Note that evoked, induced, and resting power values were converted to a logarithmic scale for the statistical analysis.

In group comparisons between the NC and SZ groups, we used cluster‐based permutation *t* tests to identify electrodes that showed significant group differences in evoked power and PLF.^51^ For induced and resting power, group differences in specific combinations of electrodes and frequency bins (electrode‐frequency bins) were explored. This exploratory approach was chosen instead of focusing on a few specific electrodes in order to avoid potential selection bias and to properly account for multiple comparisons. The detailed procedures for this test were as follows: First, unpaired *t* values were calculated to examine group differences in the respective measures at each electrode for the evoked power and PLF or at each electrode‐frequency bin for the induced and resting power. Electrodes or electrode‐frequency bins with uncorrected *P*‐values <0.001 were then clustered. For this clustering step, electrode and frequency adjacencies were determined with the MNE ‐Python functions ‘mne.channels.find_ch_adjacency’ (mne.tools/dev/api/statistics.html#) and ‘mne.stats.combine_adjacency’ (mne.tools/stable/generated/mne.stats.combine_adjacency.html#). Second, the cluster‐level statistics were obtained by summing the *t* values (Tsum) within each identified cluster. We then performed a permutation test on these cluster‐level statistics by creating 1000 surrogate Tsum values from 1000 random partitions of all the subjects' data between groups and defining the Tsum as the largest absolute sum of the *t* values across all clusters for each partition. A cluster‐level *P* value was delivered by comparing the observed cluster‐level statistics with the histogram of the surrogate Tsum values. Finally, we identified significant group differences (cluster‐level *P* < 0.05) at electrodes or electrode‐frequency bins within the resulting clusters for each measure. After significant clusters were obtained, individual values of each measure were averaged across electrodes (for evoked power or PLF) or across electrode‐frequency bins (for induced and resting power) within each significant cluster. We analyzed the between‐group effect sizes (Cohen's *d*) based on these cluster values to compare the differential sensitivities of each measure to SZ. Since PLA involves circular data, with radians as the unit, a statistical analysis for circular data is needed. We examined group differences by performing the Watson‐Williams test^52^ on the averaged PLA values across electrodes within significant clusters identified through the cluster‐based permutation test for PLF. Cohen's *d* for PLA was calculated by applying linear z score transformations to measured values based on the means and standard deviations (SDs) in NC.

To statistically evaluate group differences in the rest–task shift of spontaneous activity over the 4–100 Hz range, we first identified the electrode‐frequency bins that had a significant difference between induced and resting power using combined data from both the NC and SZ groups *via* the cluster‐based permutation *t* test. The procedures and parameters of the cluster‐based permutation test were nearly identical to those used for group comparison of each oscillatory measure with the following exception: In this analysis, we created electrode‐frequency clusters by testing the differences between induced and resting power using paired *t* tests. The surrogate histogram of Tsum values for the permutation test was obtained by taking 1000 random partitions of all subjects' data between induced and resting power, and defining Tsum as the largest absolute sum of *t* values across clusters for each partition. Finally, significant differences between induced and resting power, i.e. the rest–task shift, within clusters were defined as cluster‐level *P*‐values less than 0.05. After the cluster‐based permutation test, repeated‐measures analyses of variance (rmANOVAs) with group (SZ *vs*. NC) as the between‐subject factor and state (resting‐state *vs*. ASSR‐state) as the within‐subject factor were conducted on the mean PSDs of electrode‐frequency bins within the significant clusters. For clusters in which significant group‐by‐state interactions were observed in the rmANOVA, *post hoc* analyses employed paired *t*‐tests to compare state differences within each group and unpaired *t*‐tests to compare group differences within each state. The *P‐*values of multiple comparison *t*‐tests were adjusted using Bonferroni correction (α = 0.05/4).

The partial least squares correlation (PLSC) was performed using the myPLS toolbox (https://github.com/danizoeller/myPLS) to explore multivariate patterns of correlation between EEG oscillatory activity and clinical symptoms in SZ patients. PLSC is a data‐driven multivariate statistical technique used to identify the latent components that express the maximal covariance of two multidimensional matrixes.^53^ In the PLSC analysis, evoked power and PLF at all electrodes and induced power and resting power at all electrode‐frequency bins were stored in a matrix (X). The PANSS five‐factor scores were stored in another matrix (Y). A cross‐covariance matrix, R, was computed between X and Y, and then latent components composed of three low‐dimensional matrixes (U, S, and V) were derived *via* singular value decomposition to R. Here, U and V are singular vectors termed EEG and PANSS saliences, reflecting the contribution of the original variables to the latent components, whereas S is a diagonal matrix containing the singular values. To assess the statistical significance of each latent component, a permutation test (1000 times) was conducted to generate a surrogate histogram of all singular values, with the significance level set to *P* <0.05. From the X and Y matrices and their corresponding saliences (U and V), we computed EEG or PANSS loadings, indicating the contribution of each EEG measure and each PANSS subscore to the multidimensional associations of all EEG measures and all PANSS subscores. Finally, the stability of each EEG or PANSS loading was evaluated using the bootstrap method (1000 iterations). The bootstrap ratio was calculated for each EEG or PANSS loading by dividing the bootstrap‐estimated mean of each loading by its bootstrap‐estimated standard deviation. EEG and PANSS loadings with a bootstrap ratio exceeding ±1.96 were deemed statistically significant. Possible effects of antipsychotics on each EEG measure were also evaluated by correlating chlorpromazine (CPZ)‐equivalent doses with the cluster‐level values of EEG measures that showed significant group differences (PLF, induced power, and resting power).^54^ In addition, to examine the relatiionship between gamma‐band stimulus‐evoked and non‐phase‐locked activities, we calculated Spearman's rank correlations of cluster PLF with cluster induced power at frequencies above the gamma‐band range separately for each group. The Bonferroni correction was applied for multiple testings of correlations according to the number of combinations in each test (CPZ *vs.* EEG measures: α = 0.05/5; PLF *vs.* induced power: α = 0.05/2).

## Results

### Group comparison of 40 Hz stimulus‐evoked measures and multi‐frequency spontaneous measures

**Fig. 2:**
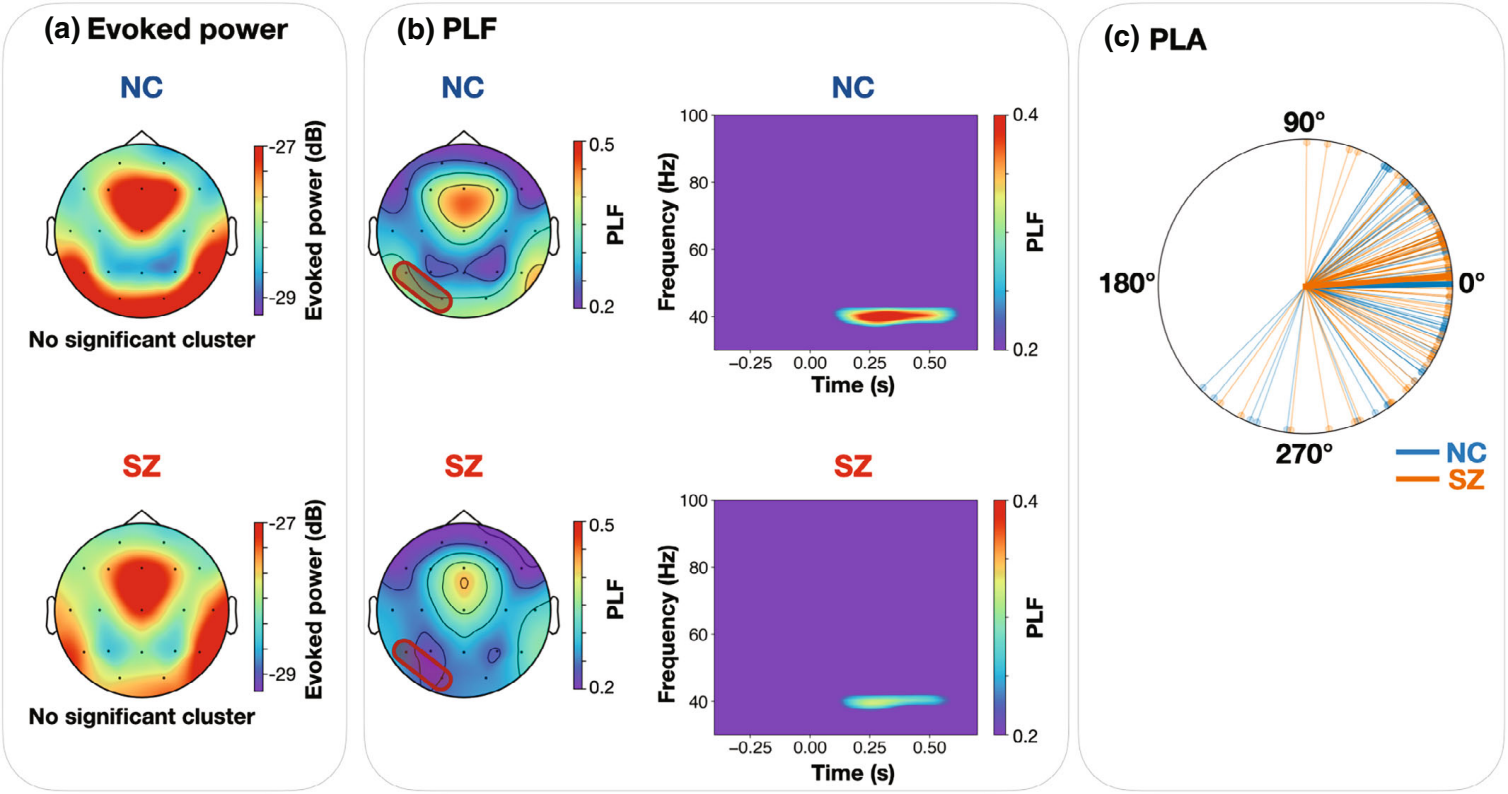
Group comparisons of 40 Hz stimulus‐evoked measures during an auditory steady‐state response (ASSR) stimulation between neurotypical controls (NC) and schizophrenia patients (SZ). (a) The topography maps of the evoked power for each group (NC, upper; SZ, lower). (b) The left panels display topography maps of the phase‐locking factor (PLF) for NC (upper) and SZ (lower) groups. The electrode cluster showing a group difference (P7 and O1) was outlined in red. The right panels present group‐averaged time‐frequency maps of the PLF at electrodes within the significant cluster. (c) Circular plot of the individual phase‐locking angles (PLAs) and group comparisons between the NC and SZ.

Figure 2a and the left column of Fig. 2b display topography maps of the average evoked power and PLF across target time‐frequency areas for each group. Cluster‐based permutation *t* tests identified one cluster containing the P7 and O1 electrodes for PLF (cluster *P* = 0.001), while no significant cluster was found for evoked power. The right column of Fig. 2b presents group‐averaged time‐frequency maps of PLF, obtained by averaging across electrodes within the significant cluster. The 40 Hz PLF was lower in SZ patients than in NC (mean [SD], 0.330 [0.127] *vs*. 0.252 [0.096]). A circular plot of the individual and group‐averaged PLA values is shown in Fig. 2c. The Watson‐Williams test revealed no significant group difference (*F*_1,132_ = 0.092, *P* = 0.762).

**Fig. 3:**
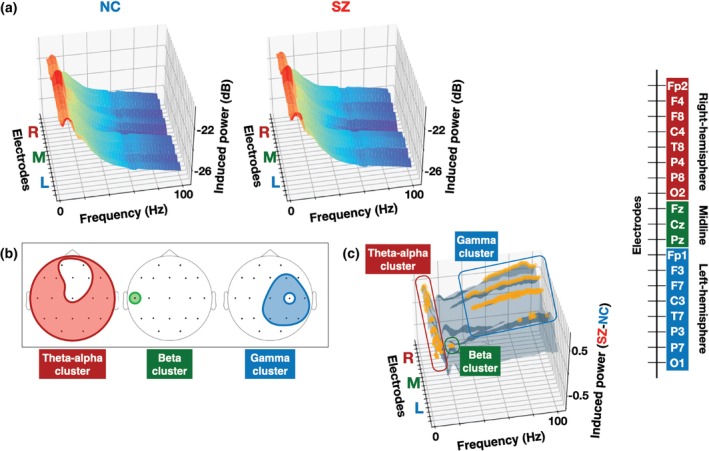
Group comparison of frequency spectra of induced power between neurotypical controls (NC) and schizophrenia patients (SZ). (a) Three‐dimensional (3D) surface plots of induced power as functions of electrode and frequency for NC (left) and SZ (right). (b) The scalp locations of the electrodes within the electrode‐frequency clusters showing the significant group difference in induced power (theta‐alpha cluster: red; beta cluster: green; gamma cluster: blue) (c) 3D surface plots of group differences (SZ – NC) of induced power as functions of electrode and frequency. The electrode‐frequency bins within each significant cluster (outlined by the same color as on the corresponding scalp maps (b)) are marked with orange dots. The electrode order of the electrode axis in all 3D surface plots is shown in the right bar.

**Fig. 4:**
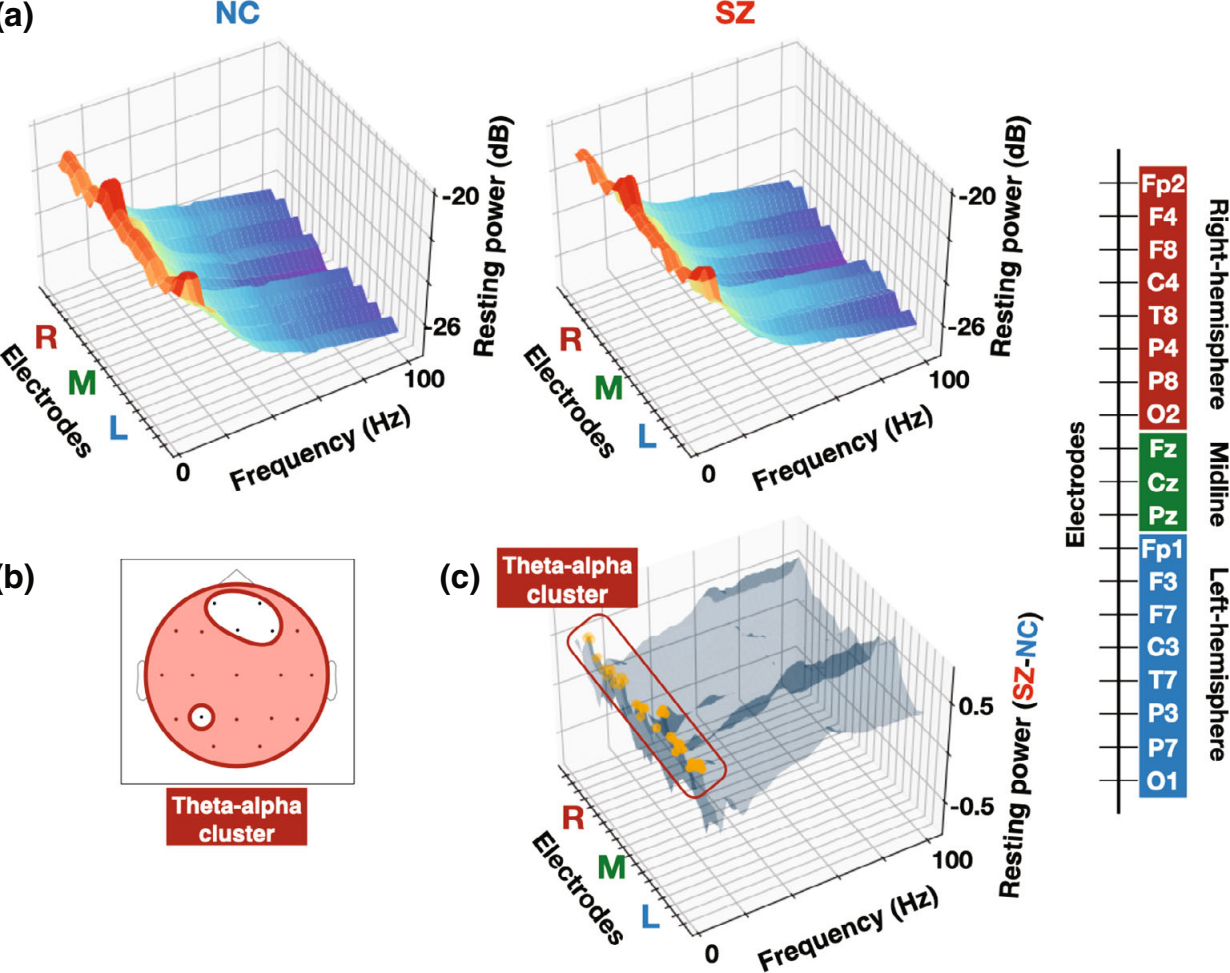
Group comparison of frequency spectra of resting power between neurotypical controls (NC) and schizophrenia patients (SZ). (a) Three‐dimensional (3D) surface plots of resting power as functions of electrode and frequency for NC (left) and SZ (right). (b) The scalp locations of the electrodes within the electrode‐frequency clusters showing the significant group difference in resting power (a theta‐alpha cluster: red). (c) 3D surface plots of group differences (SZ – NC) of resting power as functions of electrode and frequency. The electrode‐frequency bins within each significant cluster (outlined by the same color as on the corresponding scalp maps (b)) are marked with orange dots. The electrode order of the electrode axis in all 3D surface plots is shown in the right bar.

Figure 3a displays three‐dimensional (3D) surface plots of induced power as functions of electrode and frequency separately for each group. As illustrated in Fig. 3b,c, the cluster‐based permutation *t* tests on induced power identified three significant electrode‐frequency clusters: (1) a theta‐alpha band cluster (cluster *P* = 0.006; 4–10 Hz) spanning bilateral frontal, temporal, parietal, and occipital electrodes (F8, C4, T8, P4, P8, O2, Pz, F3, F7, C3, T7, P3, P7, O1); (2) a beta‐band cluster (cluster *P* = 0.045; 17–20 Hz) restricted to the left mid‐temporal electrode T7; and (3) a gamma‐band cluster (cluster *P* = 0.001; 39–100 Hz) covering frontal, temporal, and parietal region of the right hemisphere (F4, T8, P4, P8) with a midline extension to Cz and Pz. In all clusters, SZ patients exhibited higher induced power than NC (theta‐alpha band cluster: mean [SD], −21.486 [0.790] *vs*. −20.910 [0.805]; beta‐band cluster: mean [SD], −23.057 [0.815] *vs*. −22.598 [0.731]; gamma‐band cluster: mean [SD], −26.441 [0.604] *vs*. −25.917 [0.798]). Figure 4a displays 3D surface plots of resting power. The cluster‐based permutation t test identified a single theta‐alpha band cluster (cluster *P* = 0.004; 4–8 Hz) extending bilaterally across frontal, temporal, parietal, and occipital electrodes (F8, C4, T8, P4, P8, O2, Cz, Pz, F3, F7, C3, T7, P7, O1) (Fig. 4b,c). Within this cluster, SZ patients exhibited higher resting power than NC (mean [SD], −21.431 [0.863] *vs*. −20.831 [0.824]). The detailed electrodes and frequencies in each cluster of induced and resting power are shown in Supplementary Table S1.

We then compared the differential sensitivity to SZ among the measures that showed a significant group difference. Among all those measures, the gamma‐band cluster of induced power showed the largest effect size (*d* = 0.745) compared to that of any other measure (40 Hz PLF: *d* = 0.703, theta‐alpha band cluster of induced power: *d* = 0.728; beta‐band cluster of induced power: *d* = 0.599; theta‐alpha band cluster of resting power: *d* = 0.716).

### Relationships among measures with significant group differences and their correlations with clinical symptoms

No significant correlation was observed between the 40 Hz PLF and gamma‐band induced power in either SZ patients or NC (NC: *rho* = −0.259, *P* = 0.036; SZ: *rho* = −0.159, *P* = 0.196). The PLSC analysis revealed no significant latent relationships between EEG measures and PANSS five‐factor scores in SZ patients. Correlations between CPZ‐equivalent doses and the cluster‐level values of EEG measures were also non‐significant (40 Hz PLF: *rho* = −0.008, *P* = 0.951; theta‐alpha band cluster of induced power: *rho* = 0.191, *P* = 0.128; beta‐band cluster of induced power: *rho* = −0.092, *P* = 0.467; gamma‐band cluster of induced power: *rho* = −0.240, *P* = 0.055; theta‐alpha band cluster of resting power: *rho* = 0.130, *P* = 0.303). Finally, no significant correlation was observed between the 40 Hz PLF and gamma‐band induced power in either SZ patients or NC (NC: *rho* = −0.259, *P* = 0.036; SZ: *rho* = −0.159, *P* = 0.196).

### Group comparison of rest–task shift in spontaneous activity

**Fig. 5:**
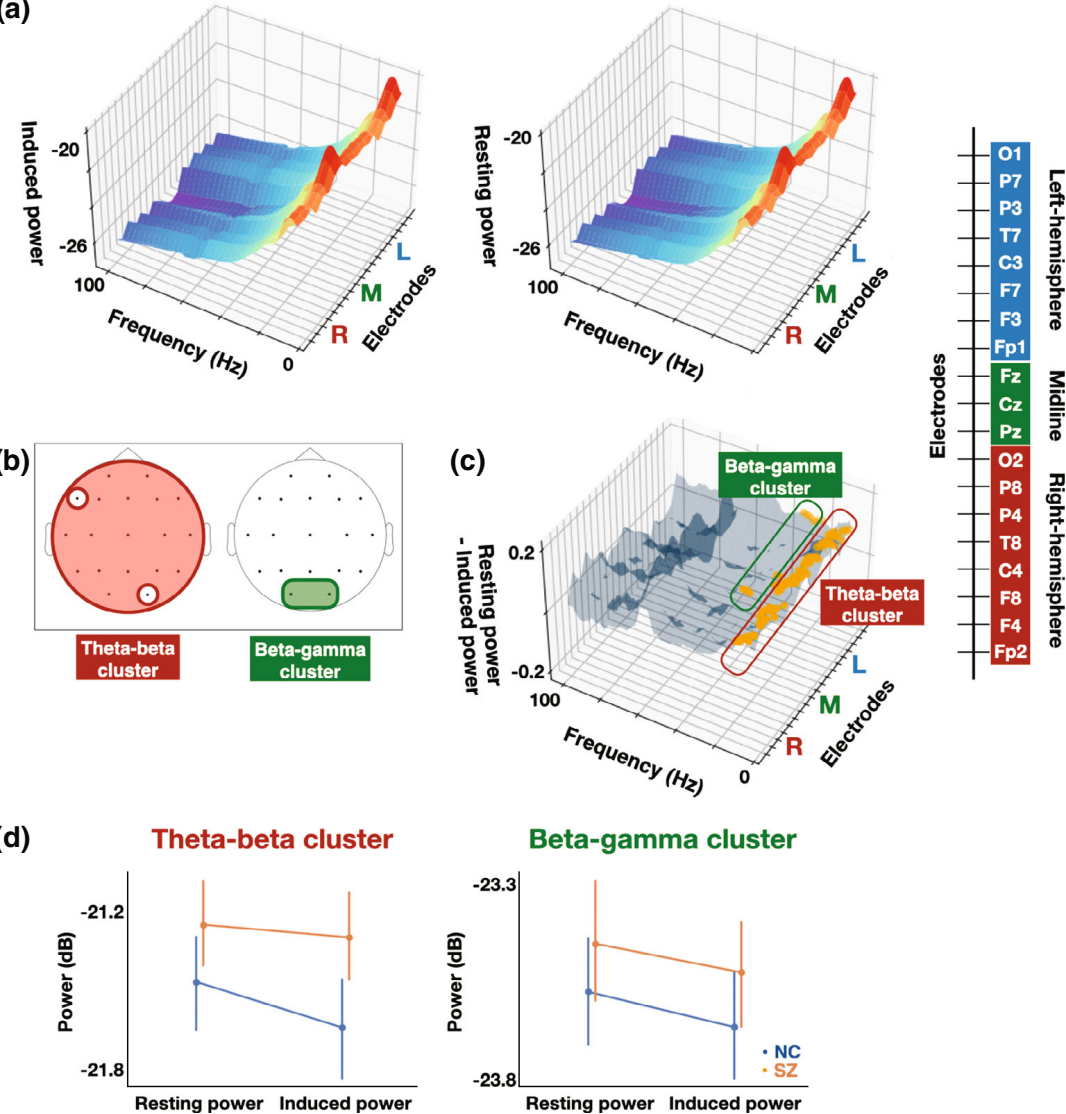
Comparisons of rest–task shifts in spontaneous power between neurotypical controls (NC) and schizophrenia patients (SZ). (a) Three‐dimensional (3D) surface plots of mean induced power (left) and resting power (right) across both groups as functions of electrode and frequency. (b) The scalp locations of the electrodes within the electrode‐frequency clusters showing the significant rest–task shifts (a theta‐beta cluster: red; a beta‐gamma cluster: green). (c) 3D surface plots of overall rest–task shifts (resting power – induced power) as functions of electrode and frequency. The electrode‐frequency bins within each significant cluster (outlined by the same color as on the scalp map (b)) are marked with orange dots. The electrode order of the electrode axis in all 3D surface plots is shown in the right bar. (d) Group comparisons of induced power and resting power in each electrode‐frequency cluster (a theta‐beta cluster: left panel; a beta‐gamma cluster: right panel). The blue lines and dots represent NC data, whereas the orange lines and dots represent SZ data. Error bars represent ± 1 standard error of the mean for each group.

Figure 5a displays 3D surface plots of overall mean induced and resting power as functions of electrode and frequency. As shown in Fig. 5b and c, we identified two electrode‐frequency clusters that exhibited significant rest–task shifts: (1) a theta‐beta band cluster (cluster *P* = 0.003; 5–23 Hz) spanning bilateral frontal, temporal, parietal, and occipital electrodes (Fp2, F4, F8, C4, T8, P4, P8, Fz, Cz, Pz, Fp1, F3, C3, T7, P3, P7, O1), and (2) a beta‐gamma band cluster (cluster *P* = 0.013; 23–32 Hz) confined to the occipital electrodes (O1 and O2). The detailed electrodes and frequencies within each significant cluster are listed in Supplementary Table S2, which shows the rest–task shift was observed mainly in the alpha‐band within the theta‐beta band cluster. The rmANOVA for PSDs within the theta‐beta band cluster (Fig. 5d, left panel) revealed significant main effects of group (*F*_1,132_ = 5.017, *P* = 0.027) and state (*F*_1,132_ = 20.869, *P* < 0.001), as well as a significant group‐by‐state interaction (*F*_1,132_ = 6.681, *P* = 0.011). *Post hoc* tests confirmed that, within the theta‐beta band cluster, induced power was significantly lower than resting power in the NC group (*t*_65_ = −4.592, *P* < 0.001), but not in the SZ group (*t*_67_ = −1.569, *P* = 0.121). Moreover, the SZ group also exhibited significantly higher induced power compared to the NC group (*t*_132_ = −2.682, *P* = 0.008), whereas no significant group difference was observed for resting power (*t*_132_ = −1.712, *P* = 0.089). In contrast, rmANOVA for PSDs within the beta‐gamma band cluster (Fig. 5d, right panel) revealed no significant group‐by‐state interaction (*F*_1,132_ = 0.168, *P* = 0.682) or main effect of group (*F*_1,132_ = 1.715, *P* = 0.193), although a significant main effect of state was detected (*F*_1,132_ = 15.872, *P* < 0.001). Specifically, within this cluster, induced power was lower than resting power across both groups (mean [SD], −21.469 [0.751] *vs*. −21.360 [0.734]).

## Discussion

Two main findings were produced in this study. First, we replicated previously reported 40 Hz ASSR abnormalities and demonstrated the high discriminability of gamma‐band induced power in differentiating SZ patients from NC. Second, a reduced rest–task shift in SZ patients was observed mainly in the alpha‐band. Together, these findings highlight the utility of the ASSR paradigm in detecting both altered 40 Hz stimulus‐evoked and multi‐frequency spontaneous activities, and suggest that disturbed state‐dependent modulation of alpha‐band spontaneous activity underlies the pathophysiology of SZ.

### Stimulus phase‐locking deficits in SZ patients

The reduced 40 Hz PLF during ASSR stimulation in SZ patients is consistent with many previous studies,^30^, ^31^, ^32^ indicating disturbances in the accurate temporal coordination of gamma‐band activity to sensory inputs in the neural circuits of SZ patients. On the other hand, while the 40 Hz evoked power is considered to be as robust a finding in SZ patients as PLF,^30^, ^31^, ^32^ our study did not replicate a decrease in that measure. The reason for the discrepant findings between evoked power and PLF*—*both stimulus‐evoked measures derived from the ASSR paradigm*—*remains unclear; however, one plausible explanation has been proposed by Hirano *et al*.^23^ They noted that the calculation of evoked power is inherently influenced by residual, non‐stimulus‐related components that cannot be completely eliminated from EEG signals. When gamma‐band induced activity is elevated, as confirmed in a previous^23^ and the present study, these residual components may obscure an actual reduction in stimulus‐locked gamma amplitude. In contrast, PLF is computed from unit‐length phase vectors and is therefore unaffected by the confounding influence of elevated spontaneous power that can distort evoked power measurements. This methodological distinction may account for the greater sensitivity of PLF relative to evoked power in detecting aberrant gamma‐band phase synchronization during ASSR stimulation in SZ patients.

The cluster‐based permutation *t* test revealed significant differences in PLF between SZ patients and NC over the left parieto‐occipital region. In most previous ASSR studies using EEG,^27^, ^29^, ^55^ time‐frequency data from the electrodes around Fz and Cz have commonly been used for sensor‐level analysis of stimulus‐evoked measures. This approach was based on the findings of Kwon *et al*.,^25^ who reported that Fz and adjacent electrodes presented the largest 40 Hz ASSR. However, there is no definitive evidence that evaluating gamma‐band ASSR measurements at electrodes with the strongest response is optimal for detecting gamma‐band oscillatory deficits in SZ patients. Recent studies have shown that the signal sources elicited by 40 Hz ASSR stimulation are distributed across broad cortical and subcortical regions.^56^, ^57^, ^58^, ^59^, ^60^ Notably, Farahani *et al*.^56^ and Tada *et al*.^59^ identified a 40 Hz ASSR signal source in the parietal lobe, close to the regions where altered 40 Hz ASSRs were observed in SZ patients in this study. Further exploratory studies are needed to clarify the pathophysiological significance of 40 Hz stimulus‐evoked activity in the parieto‐occipital region in SZ patients.

We anticipated a significant group difference in PLA during ASSR stimulation, as Roach *et al*.^33^ reported that 40 Hz PLA showed a larger effect size in SZ‐NC comparisons than evoked power or PLF did. However, we did not observe a significant group difference in PLA. This discrepancy suggests that 40 Hz PLA during the ASSR paradigm may not be a robust marker of SZ. Given the limited number of reports assessing PLA in SZ patients, further studies are needed to investigate the usefulness of PLA as a biomarker.^22^, ^33^, ^34^, ^61^, ^62^

### Increased multi‐frequency spontaneous power in SZ patients

Our exploratory, multi‐frequency analysis revealed two distinct patterns of spontaneous power enhancement in SZ patients: a state‐independent, scalp‐wide elevation in low‐frequency bands (theta and slow alpha) activity, and focal increase in high‐frequency bands (beta and gamma) activity confined to the ASSR‐state. These spatial and state profiles across multiple frequency bands indicate that spontaneous brain rhythms in SZ exhibit a band‐ and state‐specific dysregulation.

SZ patients exhibited elevated low‐frequency spontaneous power during both the ASSR and resting states in almost the entire scalp region. These widespread low‐frequency abnormalities align with the pathophysiological mechanisms proposed by the thalamocortical dysrhythmia (TCD) framework, which attributes increased low‐frequency activity due to a decelerated thalamic pacemaker.^63^, ^64^ In this model, increased inhibitory control (or reduced excitatory drive) within the thalamus slows its intrinsic alpha‐band rhythm, and the resulting low‐frequency oscillations are propagated to widespread cortical regions *via* extensive thalamocortical projections.^65^, ^66^ A recent EEG study in SZ has indeed documented a generalized slowing of rhythmic activity, supporting the applicability of this hypothesis in SZ pathophysiology.^67^ Within the TCD framework, the widespread increase in low‐frequency spontaneous power suggests that disrupted thalamic drive may constitute a core mechanism underlying oscillatory dysfunction in SZ. In this context, the state‐independency of increased low‐frequency activity indicates that the impaired thalamic pacemaker in SZ operates regardless of with or without sensory inputs.

Our finding of elevated high‐frequency induced power during ASSR stimulation in SZ aligns with previous findings.^23^, ^35^ Interestingly, the TCD hypothesis also provides a plausible explanation for the localized increase in high‐frequency spontaneous power in SZ patients. In this model, increased low‐frequency oscillations driven by the decelerated thalamic pacemaker propagate widely across the cortex and result in ectopic fast‐band bursts as the edge effect by reducing local lateral inhibition.^66^ In terms of spatial and state dependency, the enhancement of high‐frequency induced power was prominent within a right‐hemisphere gamma‐band cluster only during the ASSR‐state. This state‐dependency is consistent with previous reports showing that spontaneous gamma‐band activity in SZ increases during the ASSR‐state but not during the resting‐state.^23^, ^44^, ^68^ From the perspective of the TCD model, this right‐hemisphere gamma‐band cluster may represent the edge effect further amplified by auditory drive in SZ. Human 40 Hz ASSR is typically stronger in the right‐hemisphere,^57^, ^69^ suggesting that the right‐lateralized network possesses an intrinsically higher excitability to external inputs. Accordingly, reduced inhibition on this right‐lateralized circuit due to TCD may explain the spatial‐dependency of increased gamma‐band spontaneous power in SZ patients.

The effect size analysis further demonstrated that gamma‐band induced power offered the highest sensitivity in differentiating SZ patients from NC across all EEG measures assessed in this study. Considering that gamma‐band induced power has demonstrated excellent long‐term test–retest reliability,^55^ the increase in gamma‐band induced power during ASSR stimulation may serve as a robust neurophysiological marker for differentiating SZ patients from NC. However, a limited number of ASSR studies,^23^, ^35^, ^44^ including animal model studies,^70^ have examined gamma‐band induced power. Taken together, our exploratory, data‐driven findings support the notion that gamma‐band induced power could detect intrinsic oscillatory abnormalities in SZ.

### An aberrant rest–task shift in alpha‐band activity in SZ

Although we expected to find a rest–task shift in gamma‐band activity because we used a 40 Hz ASSR paradigm that enhanced gamma‐band stimulus‐evoked activity, significant rest–task shifts were instead observed mainly in alpha‐band activity. To our knowledge, this is the first study to demonstrate abnormal rest–task shifts in neural oscillations in SZ patients *via* the ASSR paradigm. We discuss the reduced rest–task shift in SZ patients in terms of alpha‐band oscillatory functions. Alpha‐band activity is considered to play a proactive role not only in cortical idling during the physiological arousal state^71^, ^72^ but also in top‐down control of attention for sensory and cognitive processing.^73^, ^74^, ^75^, ^76^ Taking these roles into account, it is conceivable that an impaired rest–task shift in SZ patients can be attributed to alpha‐band dysfunction in top‐down attentional control. In addition to our findings, a previous study reported a reduced shift in alpha‐band (10–12 Hz) activity between resting‐state and the P300 task in SZ patients.^77^ While that study evaluated the rest–task shift by comparing resting‐state (spontaneous) power with task‐related (P300) power, we focused on state‐dependent modulation of spontaneous power between the ASSR and resting states. Aberrant alpha‐band rest–task shifts in spontaneous activity in SZ patients may reflect impaired regulation of internal attention rather than external attention.

### Limitations

Although we obtained meaningful results regarding 40 Hz ASSR deficits and impaired alpha‐band rest–task shifts in SZ patients, several limitations should be acknowledged. First, SZ patients were treated with various types of antipsychotics. Consequently, we could not evaluate oscillatory activity independently of the potential effects of antipsychotics. In the present study, we did not find significant correlations between the CPZ‐equivalent doses and the cluster‐level values of EEG measures entered into the PLSC analysis. However, given the complex effects of antipsychotics on neural oscillations,^78^, ^79^ we cannot rule out pharmacological effects on the present study's results. Second, our investigation focused on a limited number of EEG indices. Recently, it was proposed that the complex and heterogeneous pathophysiology of SZ cannot be fully revealed by examining only a limited set of neural indices. Gordillo *et al*.^80^ explored 194 EEG measurements in SZ patients and reported very weak correlations among the EEG measurements that exhibited significant differences between SZ patients and NC. Notably, Clementz *et al*.^81^ demonstrated that various types of EEG abnormalities can be used to distinguish psychotic patients with neurobiological subtypes that do not strictly align with DSM diagnostic criteria. Therefore, considering that distinct EEG features may reflect diverse aspects of SZ pathology, it would be prudent to examine a larger number of EEG features to elucidate the underlying neural mechanisms of the highly heterogeneous pathology of SZ.

## Conclusion

We confirmed that elevated gamma‐band spontaneous power can be used to effectively detect intrinsic oscillatory abnormalities in SZ patients. Furthermore, our results also revealed that a reduced rest–task shift in alpha‐band spontaneous activity may represent a core feature of SZ. Collectively, these results highlight the pathophysiological significance of state‐dependent neural oscillatory modulation in SZ patients. Future studies with larger EEG datasets will further elucidate the clinical applicability of abnormal state‐dependent activity in SZ patients.

## Disclosure statement

Yoji Hirano is an Associate Editor and Art Advisor of Psychiatry and Clinical Neurosciences and a co‐author of this article. To minimize bias, he was excluded from all editorial decision‐making related to the acceptance of this article for publication. The authors declare that the research was conducted in the absence of any commercial or financial relationships that could be construed as potential conflicts of interest.

## Author contributions

Conceptualization: R.M., S.T., T.O., and Y.H.; Formal analysis: R.M., and S.T.; Funding acquisition: S.T., T.O. and Y.H.; Investigation: R.M., S.T., T.O., and Y.H.; Methodology: R.M., S.T., and Y.H.; Project administration: S.T., T.O., and Y.H.; Resources: S.T., T.O., and Y.H.; Supervision: S.T., S.H., T.N., T.O., and Y.H.; Validation: S.T., Y.S., Y.T., T.M., and Y.H.; Visualization: R.M., S.T. and Y.H.; Writing – original draft: R.M., S.T., and Y.H.; Writing – review & editing: R.M., S.T., S.H., Y.S., Y.T., T.M., T.N., T.O., and Y.H.

## Supporting information


**Table S1.** The specific electrodes and frequencies in each cluster that showed significant group differences in the cluster‐based permutation *t*‐test.
**Table S2.** The specific electrodes and frequencies in each cluster that showed significant rest‐task shift in the cluster‐based permutation *t* test.

## Data Availability

The data used in this study are not available publicly owing to the restriction on sharing research data from the Research Ethics Committee but are available upon request to the corresponding author.
