# Supplementary material for: Abnormal spontaneous activity and rest–task shift in schizophrenia
Source: Psychiatry Clin Neurosci. 2025 Aug 12;79(10):697–706. doi: 10.1111/pcn.13880 (PMC12498128; doi:10.1111/pcn.13880)
Supplement: Supplementary file 1 — Table S1. The specific electrodes and frequencies in each cluster that showed significant group differences in the cluster‐based permutation t‐test. Table S2. The specific electrodes and frequencies in each cluster that showed significant rest‐task shift in the cluster‐based permutation t test. [file PCN-79-697-s001.docx]

| **Supplementary table 1.** | | | | | | | |
| --- | --- | --- | --- | --- | --- | --- | --- |
| The specific electrodes and frequencies in each cluster that showed significant group differences in the cluster‑based permutation *t* test. | | | | | | | |
| Induced power | | | | | | Resting power | |
| Theta-alpha cluster | | Beta cluster | | Gamma cluster | | Theta-alpha cluster | |
| Electrode | Frequency (Hz) | Electrode | Frequency (Hz) | Electrode | Frequency (Hz) | Electrode | Frequency (Hz) |
| F8 | 4-8 | T7 | 17-20 | F4 | 57-100 | F8 | 4-6 |
| C4 | 5-10 |  |  | T8 | 39-50, 51-52, 55-100 | C4 | 5-7 |
| T8 | 6-8 |  |  | P4 | 43-49, 61-100 | T8 | 5-6 |
| P4 | 4-8 |  |  | P8 | 43-100 | P4 | 4-7 |
| P8 | 4-7 |  |  | Cz | 87-90 | P8 | 4-6 |
| O2 | 4-8 |  |  | Pz | 77-79, 86-90, 99-100 | O2 | 4-7 |
| Pz | 5-8 |  |  |  |  | Cz | 5-7 |
| F3 | 6-7 |  |  |  |  | Pz | 4-7 |
| F7 | 4-9 |  |  |  |  | F3 | 5-6 |
| C3 | 5-8 |  |  |  |  | F7 | 4-7 |
| F7 | 4-9 |  |  |  |  | C3 | 5-7 |
| C3 | 5-8 |  |  |  |  | T7 | 4-8 |
| T7 | 4-10 |  |  |  |  | P7 | 4-6 |
| P3 | 6-7 |  |  |  |  | O1 | 4-7 |
| P7 | 4-7 |  |  |  |  |  |  |
| O1 | 4-9 |  |  |  |  |  |  |

| **Supplementary table 2.** | | | |
| --- | --- | --- | --- |
| The specific electrodes and frequencies in each cluster that showed significant rest－task shift in the cluster‑based permutation *t* test. | | | |
| Rest-task shift | | | |
| Theta-beta cluster | | Beta-gamma cluster | |
| Electrode | Frequency (Hz) | Electrode | Frequency (Hz) |
| Fp2 | 7-10, 11-14 | O1 | 23-32 |
| F4 | 7-16 | O2 | 27-31 |
| F8 | 7-9 |  |  |
| C4 | 7-11 |  |  |
| T8 | 6-14 |  |  |
| P4 | 8-10, 10-14 |  |  |
| P8 | 7-8 |  |  |
| Fz | 7-17 |  |  |
| Cz | 7-20 |  |  |
| Pz | 7-12, 20-23 |  |  |
| Fp1 | 8-10, 11-12 |  |  |
| F3 | 7-15 |  |  |
| C3 | 5-14 |  |  |
| T7 | 7-13 |  |  |
| P3 | 6-14 |  |  |
| P7 | 5-14 |  |  |
| O1 | 21-22 |  |  |
